# Sex in Cheese: Evidence for Sexuality in the Fungus *Penicillium roqueforti*

**DOI:** 10.1371/journal.pone.0049665

**Published:** 2012-11-21

**Authors:** Jeanne Ropars, Joëlle Dupont, Eric Fontanillas, Ricardo C. Rodríguez de la Vega, Fabienne Malagnac, Monika Coton, Tatiana Giraud, Manuela López-Villavicencio

**Affiliations:** 1 Department Systématique et Evolution, Origine, Structure, Evolution de la Biodiversité, UMR 7205 CNRS-MNHN, Muséum National d’Histoire Naturelle, Paris, France; 2 Ecologie, Systématique et Evolution, Université Paris-Sud, Orsay, France; 3 Université Paris-Sud, Institut de Génétique et Microbiologie, UMR 8621, Orsay, France; 4 Université de Brest, UEB, Laboratoire Universitaire de Biodiversité et d’Ecologie Microbienne EA 3882, IFR148 SFR ScInBioS, ESMISAB, Technopôle Brest-Iroise, Plouzané, France; 5 Université Paris Diderot, Sorbonne Paris Cité, UFR des Sciences du Vivant, Paris, France; University of Ottawa, Canada

## Abstract

Although most eukaryotes reproduce sexually at some moment of their life cycle, as much as a fifth of fungal species were thought to reproduce exclusively asexually. Nevertheless, recent studies have revealed the occurrence of sex in some of these supposedly asexual species. For industrially relevant fungi, for which inoculums are produced by clonal-subcultures since decades, the potentiality for sex is of great interest for strain improvement strategies. Here, we investigated the sexual capability of the fungus *Penicillium roqueforti*, used as starter for blue cheese production. We present indirect evidence suggesting that recombination could be occurring in this species. The screening of a large sample of strains isolated from diverse substrates throughout the world revealed the existence of individuals of both mating types, even in the very same cheese. The MAT genes, involved in fungal sexual compatibility, appeared to evolve under purifying selection, suggesting that they are still functional. The examination of the recently sequenced genome of the FM 164 cheese strain enabled the identification of the most important genes known to be involved in meiosis, which were found to be highly conserved. Linkage disequilibria were not significant among three of the six marker pairs and 11 out of the 16 possible allelic combinations were found in the dataset. Finally, the detection of signatures of repeat induced point mutations (RIP) in repeated sequences and transposable elements reinforces the conclusion that *P. roqueforti* underwent more or less recent sex events. In this species of high industrial importance, the induction of a sexual cycle would open the possibility of generating new genotypes that would be extremely useful to diversify cheese products.

## Introduction

The maintenance of sex remains one of the most fundamental questions in evolutionary biology. In sexually reproducing organisms, recombination breaks down favourable gene combinations built by past selection. Moreover, when males contribute little or no resources to their progeny, a mutation causing females to reproduce asexually is expected to thrive because its frequency doubles at each generation (which is known as the ‘twofold cost of sex’) [1]. Various additional costs associated with mating apply to more specific cases, including costs of finding and courting a mate, risks of predation, of contracting sexually transmitted diseases or parasitic genetic elements [2]. Despite these heavy costs, most eukaryotes engage in sexual recombination at least at some point in their life cycle. Sexual reproduction seems essential for the long-term persistence of species [3] and only a few long lived asexual lineages are known to persist on the long-term without sex [4], [5]. But even in some of these textbook “evolutionary scandals”, such as bdelloid rotifers or Glomeromycota fungi, cryptic sex has been suggested to exist based, for example, on the presence of functional meiotic genes [6], [7]. Sex is indeed considered to provide some advantages that would balance its costs. Empirical studies have shown that sex reduces the accumulation of deleterious mutations compared to asexual reproduction [8]. It can also lead to a more rapid adaptive response by bringing together beneficial mutations, allowing their fixation in the populations, especially in harsh environments where selection is strong [5], [9], [10].

In most multicellular eukaryotes, sexual reproduction is regulated by strict mechanisms governing which haploids can fuse at syngamy. These mechanisms called mating types, sexes or genders, control and limit the number of compatible mates in a population. Mating compatibility can be controlled for instance by dimorphic sex chromosomes in mammals, or by more polymorphic systems involving dozens to hundreds of alleles determining mating types in other groups, such as the self-incompatibility system in plants and mating types in fungi and protists.

In Ascomycota fungi, mating compatibility is regulated by two highly dissimilar idiomorphs (named as such rather than alleles due to the uncertainty of their origin by common descent) that are located at the mating type locus [11]. Ascomycota are bipolar, *i.e.* compatibility is determined by idiomorphs at a single locus [12]. MAT genes code for transcription factors that induce the production of pheromones and pheromone receptors [13] and they can also be involved in a number of mating functions related to sexual differentiation and sexual development [13]. Sexual reproduction in heterothallic Ascomycota can only occur between two haploid individuals carrying alternate idiomorphs at the MAT locus. Homothallic fungi do not need a mate being different at the MAT locus to reproduce meiotically, most often because each haploid carries both idiomorphs in its genome. Each haploid is therefore compatible with every other haploid in the population and syngamy is even possible between clonemates in a process called same-clone mating or haploid selfing [14].

Fungi are a group historically considered to present a high proportion of asexual species; a fifth of species were once thought to reproduce exclusively asexually [15]. Sex is considered to have been lost several times independently in groups such as the *Penicillium* subgenus *Biverticillium* [16], [17]. However, many of these supposedly asexual species have been found to actually reproduce sexually in nature, as evidenced by population genetics [18]–[20], or have been shown to reproduce successfully under lab conditions [21]. Other signs of sex in supposedly asexual species include footprints of Repeat Induced Point mutations (RIP), an irreversible similarity-dependent gene mutation mechanism specific to fungi, occurring only during the sexual stages on repeated sequences [22]. Finally, in some species considered to be asexual, the MAT locus has been identified (see [22] for reviews), and in some species, idiomorphs have even been found to evolve under purifying selection, as expected for functional genes [17], [23]. Such evidence suggests that sexual reproduction is still occurring in nature, or that it occurred until recently, because constraints on genes involved in sexual reproduction would be expected to be relaxed in asexual species.

Here, we investigated the possibility of sex in the ascomycete fungus *Penicillium roqueforti.* This filamentous Ascomycota is used as a starter culture for the production of most blue veined-cheeses worldwide (Roquefort in France, Danablue in Denmark, Cabrales in Spain or Gorgonzola in Italy). In contrast to other cheese fungi, *P. roqueforti* is not exclusively found in dairy environments. Indeed, its capacity to tolerate cold temperatures, low oxygen concentrations, alkali and weak acid preservatives, makes this species a common spoilage agent in refrigerated stored foods, meat, wheat products, silage and even forest soil and wood [24]–[26]. This fungus is of great economic importance, as shown by the production of 56,865 tons of blue veined cheeses in France each year [27]. In cheese industries, spores of *P. roqueforti,* bought from starter strain producers who exclusively grow the fungus by clonal subcultures, are introduced into the cheese curd. Thus, as in many biotechnological species, strains used in cheese making are clones maintained by asexual replication. Strain improvement therefore only relies on random mutagenesis, followed by screening for variants exhibiting enhanced properties of interest [11], such as growth, mycelium thickness and colonies colour or lipolytic and proteolytic actions. Creating genetic variation through mating and recombination would be of great interest. Indeed, the emergence of new genotypes is expected to be faster in sexually reproducing clades where recombination bring together new mutations appearing in different individuals [10], [28]. Sex is also known to be important for purging faster deleterious mutations that accumulate during clonal propagation [8]. Until recently, the two other species belonging to the same clade as *P. roqueforti* (the *Penicillium* series *roqueforti), i.e. P. paneum* and *P. carneum*, were also considered asexual [29]. However, a new species producing cleistothecia was discovered in 2010 and assigned to this clade on the basis of phenotypic characters, sequences and extrolite patterns [30].

We searched for footprints of sex in the species *P. roqueforti* using genomic and molecular approaches. A large collection of *P. roqueforti* strains coming from different geographical locations worldwide was screened in order to assess whether ratios of mating types were balanced, which is an indication of regular sex [31]. We used molecular markers (two microsatellites, the β-tubulin gene and the MAT locus) to look for footprints of recombination. Moreover, we took advantage of the recently sequenced genome of a *P. roqueforti* strain isolated from cheese environment (strain FM 164, Ropars et al., unpublished data) to identify a large set of genes involved in the meiotic machinery and to search for footprints of Repeat Induced Point mutation (RIP) in the genome. Finally, we analysed the selective pressures acting on mating type genes and in a subset of genes essential for meiosis in order to assess if these genes evolved under purifying selection, as expected for functional genes [23].

## Materials and Methods

### Fungal Isolates

The *P. roqueforti* FM 164 sequenced isolate used as inoculum during cheese production was used (unpublished data). A total of 126 other isolates of *P. roqueforti* coming from 14 different countries were also screened (Table S1): 43 isolates (including the FM 164 sequenced strain) from the cheese environment were provided by French producers of starter cultures and cheeses, and were designated as FM numbers to keep their origin confidential. A total of 16 strains isolated from diverse environments such as silage or the interior of refrigerators were obtained from public collections (CBS, LCP, MUCL), including the type strain (Table S1). Monospore isolation was systematically made when commercial dairy strains were received using a dilution method to guarantee the presence of pure isolated colonies. Finally, 68 other isolates were directly isolated from 38 different cheeses coming from 14 countries throughout the world and were designated as “F” numbers. The number after the “F”, from 1 to 38, corresponds to the individual cheese. These 68 “F” strains were obtained by dilution of spores in order to obtain single colonies and to isolate the different morphotypes grown for 3–5 days at 25°C on Malt Agar. For each of the 38 cheeses, morphologically different strains were selected and treated as different individuals. These individuals are labelled by a number following the one identifying the cheese (e.g. F37.1 and F37.4 are two individuals isolated from the same cheese, labelled 37).

### DNA Extraction, PCR Amplification and Sequencing

Genomic DNA was extracted from fresh mycelium of isolates listed in Table S1 grown for 3–5 days on Malt Agar. The Qiagen DNeasy Plant Mini Kit (Qiagen, Ltd. Crawley, UK) was used for extraction and purification. Strains were identified using the 5′ end of the β-tubulin gene (oligonucleotide primer set Bt2a/Bt2b [32]) in order to check that they belonged to the *P. roqueforti* species [29]. Sequences of the β-tubulin are identical but three bp localized in a non-coding region, splitting the species into two subclades. The β-tubulin sequences of *P. roqueforti* strains belonging to these two subclades had been deposited in GenBank, for example, accession numbers HQ442359.1 [30] and AY674380 [29] for one clade and AY674383.1 [29] and AY674382.1 [29] for the other.

The mating type oligonucleotide primer sets used in this study were designed in conserved regions of MAT1-1 and MAT1-2, using Primer3 online (http://www.frodo.wi.mit.edu/cgi-bin/primer3/primer3_www.cgi). In order to obtain the MAT1-1 sequence of *P. roqueforti* (Accession number JX627318 for the FM 317 strain), we used the sequences of the close relatives *P. chrysogenum* Wisconsin [33], *Eupenicillium crustaceum* [34], *P. fuscoglaucum* (Accession number JX627319) and *P. paneum* (Accession number JX627320). For MAT1-2, we used the genomic sequence of *P. roqueforti* FM 164 (Accession number JX627321). Primers sequences used were CTG-GGC-CAC-GTT-TTG-TCT-AT for MAT1-1F, ATT-GGC-CAC-TGA-GGA-ACA-TC for MAT1-1R and CDC-CCA-YCT-TCC-YCA-GAC-T for MAT1-2F, RCG-ACG-AGG-AGC-RTA-YTG-AT for MAT1-2R. All the *P. roqueforti* isolates were screened by PCR for mating type idiomorphs.

The structure of the idiomorph MAT1-2 was recovered using the genomic sequence of *P. roqueforti*.

We genotyped the strains at all available microsatellite markers [35] but only PC4 and PC13 revealed polymorphic.

PCRs were performed in 50 µl reactions, using 25 µl of template DNAs, 1.25 U of AmpliTaq DNA polymerase (Roche Molecular Systems, Inc., Branchburg, NJ, USA), 5 µl of 10X Taq DNA Polymerase buffer, 5 µl of 50% glycerol, 2 µl of 5 mM dNTPs (Eurogentec, Seraing, Belgium), 2 µl of each 10 µM primer and 50–100 ng template DNA. Amplifications were performed in a BioRad DNA Engine Peltier Thermal cycler with 35 cycles of 30 s at 94°C, 30 s at 56°C and 60 s at 72°C. The PCR program was followed by a final 7 min extension step at 72°C.

### Orthology Search of the Meiotic Toolbox

Via a BLASTP search using the best reciprocal hit method (BRH), the genome of *P. roqueforti* (strain FM 164) was screened for a previously described set of 88 *Neurospora crassa* conserved proteins involved in meiosis [36] (Table S2).

### Detection of RIP

Using the sequenced genome of *Penicillium roqueforti* FM 164, we searched for RIP-like footprints in both repeated sequences and in some transposable element families. Repeated sequences were obtained by BLASTing all predicted CDS sequences of the *P. roqueforti* genome against each other using MEGABLAST. Sequences were then clustered using the following criteria: the alignment should cover 400 bp of the query protein sequence, the identity must be greater than 80%. To detect the TE families, three known transposable elements families were selected in the *P. chrysogenum* Wisconsin 54–1255 genome available online (Genbank accession NS_000201, newly re-identified as *P. rubens,* [37]): Copia-like LTR retrotransposons belonging to the class I (RNA mediated elements retrotransposons), Tan transposons belonging to the Pogo family in the Tc1/mariner superfamily of class II (DNA mediated elements transposons), and Tc5, also belonging to the class II transposons. The transposable elements were identified in the *P. roqueforti* genome via a BLASTP search. Multiple sequence alignments were then built using ClustalW with default settings [38]. RIP involves transitions from C:G to T:A nucleotides in pairs of duplicated sequences during the dikaryotic phase between mating and meiosis, usually targeting a specific dinucelotide site. These changes in dinucleotide frequencies can be used to identify RIP-affected repeats by measuring the ratios of pre-RIP and post-RIP di-nucleotides within a set of repeated sequences, thus generating so-called RIP indices, or by identifying specific target RIP-site in alignments showing C:G to T:A polymorphism. RIP-like footprints were searched for using the software RIPCAL [39], that uses RIP indices and alignment-based methods.

### Assessing Selective Pressures

Patterns of selective pressure can be investigated using molecular evolution codon models and measuring the non-synonymous/synonymous substitution ratio (dN/dS), also referred to as ω. ω<1 indicates purifying selection, ω>1 suggests positive selection and ω = 1 relaxed selection or neutral evolution [40]–[42]. We assessed the type of selective pressures for the following *P. roqueforti* genes: 1) mating type genes, 2) the twelve genes involved almost exclusively in meiosis and 3) the *rid* gene encoding a protein required for RIP.

Codon-based multiple sequence alignments were carried out using PAL2NAL [43] on the basis of protein sequences alignment with MAFFT [44]. Selective pressures were estimated using the web server SELECTON version 2.4 [45], [46]. The server was run with the M8 model, assuming that ω values come from a mixture of a discrete beta distribution and an additional category ω_s_ ≥1 (positive selection), and compared with the M8a null model, assuming no positive selection [47], [48]. Under the “selective” model (M8), the ω ratios (dN/dS) were estimated using a bayesian approach at each site of the protein, reporting either positive selection (ω>1), neutral evolution (ω = 1) or purifying selection (ω<1).

#### 1) Selective pressures acting on mating type genes

For assessing the selective pressures acting on mating type idiomorphs two alignments, including respectively the MAT1-1 and MAT1-2 sequences, were constructed. For *P. roqueforti*, we used the entire sequence of the MAT1-2 idiomorph from the sequenced strain FM 164 and the partial sequence of the MAT1-1 idiomorph obtained by PCR from another cheese strain (FM 317 strain). We also included the MAT1-1 and MAT1-2 sequences of the homothallic species *Eupenicillium crustaceum* whose mating type genes have been described [34], of the heterothallic species *P. chrysogenum* [49] and of close relatives of *P. roqueforti* from which genomes are currently being sequenced (*P. paneum*, *P. nalgiovense*, *P. camemberti, P. fuscoglaucum* and *P. biforme*) (Table 1). These five latter species appear heterothallic, containing a single MAT idiomorph (either MAT1-1 or MAT1-2) in their genome. Because a single haploid genome was available for each species, they were only represented in one of the two alignments (Table 1 for details). Both alignments contained six species: *E. crustaceum, P. roqueforti* and *P. chrysogenum* were represented in both alignments while *P. biforme*, *P. camemberti* and P. *nalgiovense* were only included in the MAT1-1 alignment, and *P. fuscoglaucum*, *P. paneum* and *P. carneum* only in the MAT1-2 alignment (Table 1). In all the cases, intron splicing was inferred manually based on the previously characterized *E. crustaceum* and *P. chrysogenum* sequences.

**Table 1:** Strains included in either the MAT1-1 or MAT1-2 alignment for analyzing selection pressures.

Species	Isolate number	Mating type		Reference
		MAT 1-1	MAT 1-2	
*Penicillium roqueforti*	FM 164		×	Ropars et al., *in prep.*
*Penicillium roqueforti*	FM 317	×		This study
*Eupenicillium crustaceum*	CBS 244.32	×		Pöggeler et al., 2011
*Eupenicillium crustaceum*	CBS 244.32		×	Pöggeler et al., 2011
*Penicillium chrysogenum*	Wisconsin 54–1255	×		van den Berg et al., 2005
*Penicillium chrysogenum*			×	Hoff et al., 2008
*Penicillium fuscoglaucum*	FM 041	×		This study
*Penicillium paneum*	FM 227	×		This study
*Penicillium biforme*	FM 169		×	This study
*Penicillium camemberti*	FM 013		×	This study
*Penicillium nalgiovense*	FM 193		×	This study

#### 2) Divergence and selective pressures on meiotic genes

Divergence and selective pressures were investigated in a subset of 12 meiotic genes, selected among all the genes screened (Table S2) as those having a fundamental role in meiosis (“meiosis specific genes”, [50]). These 12 meiotic genes analysed were spo11 and ski8, involved in double-strand breaks formation and processing, DMC1, RAD51 and RAD54, involved in single strand invasion, mutL, MSH4 and MSH5, involved in the regulation of crossover frequency, mus50, involved in DNA repair, RAD21, rec8, and a protein required for establishment and maintenance of sister chromatid cohesion, these three latter genes being involved in chromosome cohesion. Using a BLASTN search, these genes were extracted from the 6 other sequenced genomes, i.e. *P. rubens* Wisconsin 54–1255 [33], *P. paneum, P. nalgiovense, P. camemberti, P. biforme* and *P. fuscoglaucum.* Each alignment was then constructed using ClustalW [38]. Introns and insertion/deletion events were removed manually. In order to evaluate meiotic genes divergence between *P. roqueforti* and its close relative *P. paneum*, we constructed gene trees under the maximum likelihood framework using Phyml package [51] with a Gamma distribution with 4 rate categories of across site rate variation. Trees were plotted in Seaview [52].

#### 3) Selective pressure acting on the *rid* gene

The *rid* gene, the only gene known to be required for RIP process, was searched in the *P. roqueforti* genome as well as in the six other genomes used for selection analyses. Alignments were constructed following the same method described above.

### Population Genetics Analyses

Linkage disequilibria among the four available polymorphic markers (Table S3) were computed using Genepop on the Web ([53], [54], http://genepop.curtin.edu.au/).

## Results

### Analyses of Mating-type Loci

#### 1) Structural organization

**Figure 1:**
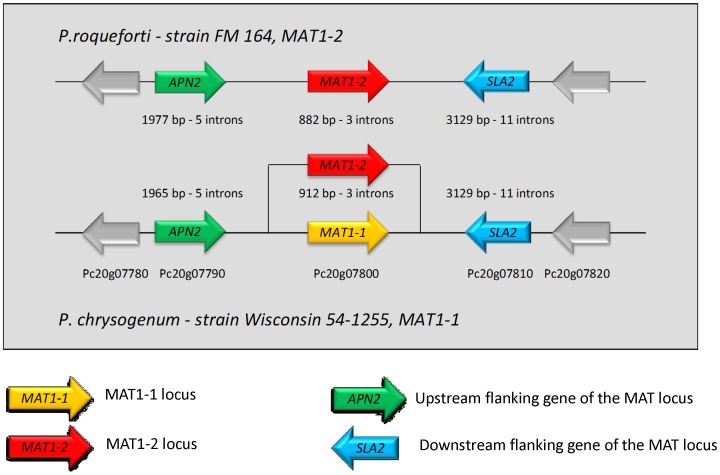
Schematic illustration of the Mating-type locus and the flanking of *Penicillium roqueforti* FM 164 (MAT1-2 strain) and its close relative *P. chrysogenum* Wisconsin 54–1255 (MAT1-1 strain). The transcriptional direction of the genes is indicated by an arrow. As the Wisconsin strain is a MAT1-1 strain, we identified the MAT1-2 gene in another *P. chrysogenum* strain (AM904545). Genes represented in both species by same colors show a nucleotide identity>80%.

As expected for heterothallic species, the sequence of *P. roqueforti* revealed the presence of a single idiomorph in the genome, corresponding to MAT1-2. The flanking regions of MAT1-2, included upstream and downstream respectively the genes *APN2* (Accession number JX627322) and *SLA2* (Accession number JX627323) (Figure 1). This structural organization is similar to that of mating-type loci in many sexually reproducing filamentous ascomycetes, and in particular in *P. chrysogenum* [34], [49], a close relative of *P. roqueforti* (Figure 1). In these two *Penicillium* species and other close relatives, such as *Aspergillus* species, the COX13 gene is lacking, while it is present in more distant ascomycete species at the MAT locus [55]. Compared to its homologue in *P. chrysogenum*, the MAT1-2 sequence contained two introns [49] and the protein was shorter by 9 amino-acids. Nucleotidic identities of the *APN2, MAT1-2* and *SLA2* coding sequences between *P. chrysogenum* and *P. roqueforti* were 85.7%, 87% and 93.2% respectively.

#### 2) Ratio of MAT idiomorphs

The screening of the 127 *P. roqueforti* isolates from diverse origins throughout the world revealed the presence of both idiomorphs in all regions, but indicated an overall significant departure from the 1∶1 expected ratio (χ^2^ = 11.98; df = 1; P = 0.001) (frequency of strains detailed in Table S1). This resulted from the higher frequency of MAT1-2 individuals among the strains directly isolated from various kinds of cheeses (“F” strains). MAT1-1 and MAT1-2 frequencies were indeed balanced in the FM strains used as inoculum during cheese production (23 strains carrying MAT1-1 and 20 MAT1-2) and in those coming from public collections isolated from non-cheese substrates (six strains carrying each MAT idiomorph). Interestingly, strains carrying alternative idiomorphs were isolated from the very same cheese in some cases (Table S1, among “F” strains).

#### 3) Selective pressure on mating type genes

**Figure 2:**
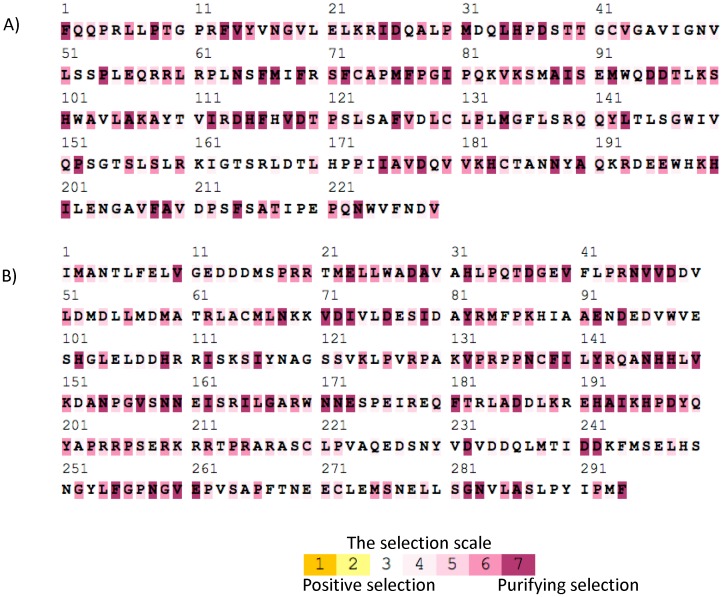
Color coded Selecton Results for a) MAT1-1 and b) MAT1-2 sequences of *Penicillium roqueforti*. Amino acids sites under purifying selection are colored in purple. Darker purple colors indicate stronger purifying selection and values of ω closer to 0. More than 70% of the sites for MAT1-1 and 50% for MAT1-2 are evolving under strong purifying selection.

The SELECTON runs did not detect footprints of positive selection in either MAT1-1 or MAT1-2. This result suggests that these genes have evolved under purifying selection or in a nearly-neutral fashion (Figure 2). The log-likelihood values for the models M8 and M8a were almost identical (MAT1-1, M8 = −3361.6 and M8a = −3361.59; MAT1-2, M8 = −3178.57 and M8a = −3178.59). The LRT for the models M8 and M8a was consequently not significant. The observation of alignments for both MAT1-1 and MAT1-2 however showed a very low signal of divergence, which could bias the selection tests. The ω values per amino acid position nevertheless suggest that recent evolution of both idiomorphs were shaped by purifying selection as a large proportion of the sites were found to be under strong purifying selection (ω<0.1) in both idiomorphs (Figure 2), *i.e.*>70% and 50% for MAT1-1 and MAT1-2, respectively.

### Meiotic Genes

All the important genes identified in the heterothallic sexually-reproducing fungus *N. crassa* as involved in meiosis were also present in *P. roqueforti* (Table S2). The divergence analysis on the subset of the 12 genes necessary for meiosis (Figure S1a-l) suggests that these genes are evolving more slowly in *P. roqueforti* than in its sister species *P. paneum*, supposedly asexual: nine of the twelve genes accumulated fewer substitutions along the *P. roqueforti* lineage than along the *P. paneum* lineage. This is confirmed by tests of positive selection (Table S4), showing that most of the sites in meiosis-related genes of *P. roqueforti* evolve under purifying selection (Table S4 for details, Figure S2 for an example) according to the site-specific test as incorporated in SELECTON. Interestingly, nine of the twelve genes contain a few sites under positive selection (assessed under the M8 model).

### Repeat Induced Point Mutation (RIP) footprints

The only gene known to be necessary for RIP process, *rid,* was identified in the *P. roqueforti* genome. The SELECTON runs did not detect any footprints of positive selection in the *rid* gene. The ω values per amino acid position suggest that recent evolution of the *rid* gene was shaped by purifying selection in a large proportion of the sites (ω<0.1).

**Figure 3:**
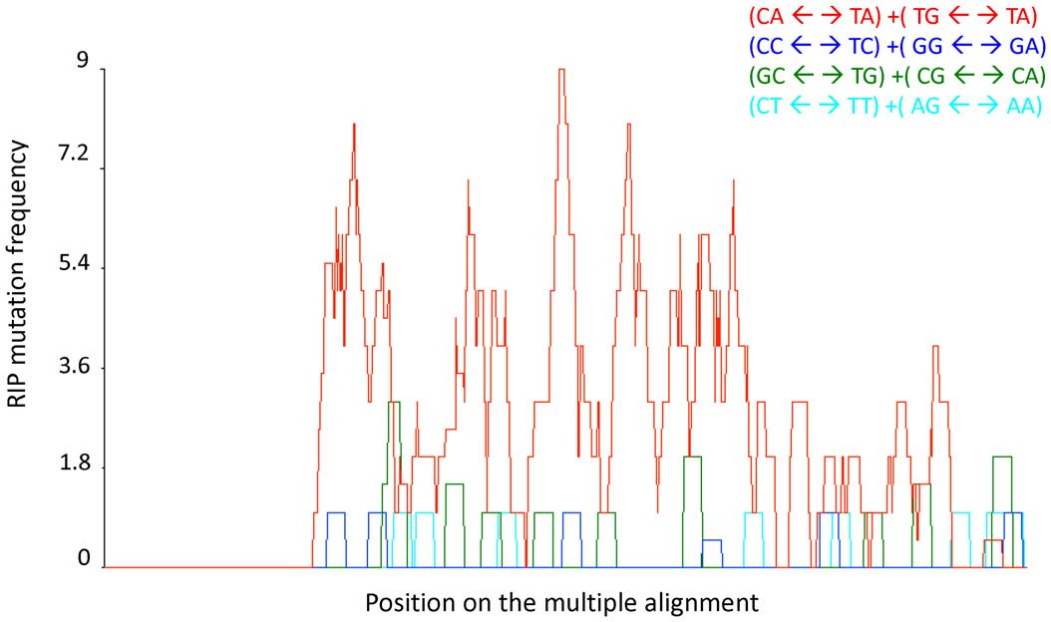
RIP mutation frequency plot over a rolling sequence window, corresponding to the multiple alignment (not shown). Nucleotide polymorphisms (against the alignment consensus, which is also the highest GC-content sequence) mostly correspond to CpA<- ->TpA or TpG<- ->TpA (red curve), as expected when RIP is acting.

RIP footprints were found in some repeated sequences in the genome of *P. roqueforti.* Among 102 clusters of genes sequence repeats showing more than 80% of identity over at least 400 bp, four displayed RIP signatures. When subjected to RIP, repeats show a characteristic substitution bias because RIP-induced mutations happen to be mostly C to T and G to A transitions. Functional gene families may however be protected against RIP. We therefore also examined transposable elements for RIP footprints. In the sequenced *P. roqueforti* strain, RIP-like footprints were detected in the three TE families analyzed for RIP-like signatures, namely Copia-like LTR retrotransposons (Figure 3), Tan transposons belonging to the Pogo family and the DNA transposons Tc5.

### Footprints of Recombination

Among all the microsatellite markers typed and the genes sequenced, only four markers revealed polymorphism: the beta-tubulin (three alleles but one was carried by a single strain and was therefore not integrated in the test of recombination), the MAT locus (two alleles) and the microsatellites PC4 and PC13 (two alleles each). Linkage disequilibria were significant among three of the six marker pairs, which was expected given the frequency of asexual propagation and the likelihood of population structure in our dataset from a worldwide collection. Nevertheless, three pairs of markers were not in significant linkage disequilibria and 11 out of the 16 possible allelic combinations were found in the dataset.

## Discussion

In many fungal species, sexual reproduction has not been observed directly in natural populations or in the laboratory because the factors needed to induce sex are unknown. For most species, direct demonstration of sexual reproduction in the lab is very laborious and time consuming because the discovery of the suitable conditions demands numerous attempts [21], [31]. Therefore, in many cases, the occurrence of sexual reproduction can only be indirectly inferred, as we do here with *P. roqueforti.*

In a great number of supposedly asexual species, population genetic analyses have nevertheless demonstrated the occurrence of recombination, such as in *Coccidioides immitis* [18], [56]. A few species appear to show an extremely clonal population structure, such as the pathogen *Penicillium marneffei* [57] (but see [58] for a recent study showing footprints of occasional sexual reproduction in this species). We did find evidence of the occurrence of recombination as 11 out of the 16 possible allelic combinations were found in our dataset, and three of the six marker pairs did not display significant linkage disequilibria.

Further evidence for the occurrence of recombination was found using the MAT locus. The MAT1-1 and MAT1-2 mating type idiomorphs were found in separate individuals of *P. roqueforti* as is generally the case for heterothallic species. The MAT1-1 and MAT1-2 idiomorphs in *P. roqueforti* did not show evidence of loss-of-function mutations and they seemed to evolve under strong purifying selection as expected in sexual species [17], [23]. While the ratio of the two MAT idiomorphs was found to be balanced amongst strains belonging to public culture collections and those used as inoculum in cheese production, individuals carrying the MAT1-2 idiomorph were overrepresented in the “F” strains directly isolated from blue cheeses. A 1∶1 ratio of mating types is considered as strong evidence that sexual reproduction is occurring in fungal populations, as drift is expected to drive one mating type to extinction in asexual species [59]–[63]. Balanced frequencies of mating types have been found in populations of species that had long been considered to be asexual before sex was successfully induced in lab conditions, e.g. in *Aspergillus fumigatus* [64]. A strong deviation from this 1∶1 ratio has however been observed in some sexual species such as *Cryptococcus neoformans* [65]. Several reasons can account for a skewed mating type ratio in sexually reproducing species. It may be that mating type genes are involved in other functions than mating regulation, such as in *C. neoformans,* where one of the mating types is associated with virulence [66]. Here, the overrepresentation of MAT1-2 individuals only among “F” strains directly isolated from cheeses could result from a bias in human selection of individuals carrying the MAT1-2 idiomorph. However, MAT1-1 and MAT1-2 were equally represented among the FM strains used as starter culture in cheese production, suggesting that they have been equally selected. Interestingly, we found *P. roqueforti* individuals carrying different idiomorphs in the very same cheese, such as in the French cheese Fourme d’Ambert (F5), in Latvian blue cheese (F28) or in the Spanish Picon Hoja cheese (F16) (see Table S1). Some industrials inoculate several strains having different desired characteristics (personal information). Our results show that both mating types can be found in the same environment, making sexual reproduction feasible and indicating the occurrence of more or less regular sex. Although no sexual structures have been observed so far for this species, it would be extremely interesting to look for some in all environments linked to cheese production.

The finding of the genes needed for sexual reproduction in the genome suggests that meiosis could be occurring in *P. roqueforti*. The presence of meiotic genes has previously been used to suggest cryptic sex in some ancient asexuals such as the bdelloid rotifers or several species of the fungal genus *Glomus* [6], [7], parasitic species in the *Giardia* genus [50] and *Trichomonas vaginalis* [67]. The analyses of divergence and selective pressures of 12 meiotic genes known to be necessary for meiosis [36], [50], revealed that these genes are more conserved in *P. roqueforti* than in the other species included in the analyses, all considered asexual, and that they seem to evolve under strong purifying selection. Nevertheless, nine of the twelve genes showed a few sites evolving under positive selection. This could result from the fine-tuning of the meiotic function in *P. roqueforti* species in its new cheese environment. Taken together, the results of the divergence analysis and the test of positive selection strongly suggest that, overall, these genes are highly conserved in order to maintain their function, but that they also continue to adapt or adapted very recently at specific sites.

Finally, evidence for RIP-like footprints was found in the *P. roqueforti* genome (Figure 3). As RIP is a similarity-dependent gene silencing mechanism specific to fungi [68], which specifically operates during the dikaryotic phase [22], RIP signatures are evidence of more or less recent sex events [69]. RIP was experimentally shown to be a currently active process in *Neurospora crassa* [22], [70]–[72], *Podospora anserina* [73], *Magnaporthe grisea* [74], *Leptosphaeria maculans* [75] and *Nectria haematococca* [76], species for which sexual reproduction can be efficiently performed in laboratory conditions. Nevertheless, *in silico* analyses showed RIP-like footprints in a large number of fungal genomes, mostly in Ascomycota, such as in several *Aspergillus* species [77]–[79], *Tuber melanosporum* [80], but also in Basidiomycota such as *Microbotryum violaceum* [81] and *Puccinia graminis* [82]. More generally these footprints have been observed in numerous species for which sexual reproduction has never been described such as *A. niger* [78], *Colletrichum cereale* [83] or *Coccidioides immitis* and *C. posadasii* [84]. As RIP has never been shown to occur without sex, RIP-like footprints are evidence for the occurrence of more or less recent sexual reproduction, although the possibility remains that sex has been lost very recently. The RIP-like footprints found in some repeated sequences and three transposable elements families of *P. roqueforti* genome are therefore further evidence suggesting that this species undergoes sexual reproduction or that sex occurred recently.

### Conclusion

In conclusion, several lines of indirect evidence suggest that *P. roqueforti* presents a sexual cycle or that it was able to reproduce sexually until recently: the screening of a large set of strains isolated from diverse substrates throughout the world revealed footprints of recombination, the presence of both idiomorphs, even in the very same cheese, and these genes appeared to evolve under purifying selection. Finally, the investigation of the recently sequenced genome FM 164 has allowed for the identification of a set of 88 proteins involved in meiosis and the detection of RIP-like footprints in repeated sequences and transposable elements. The detection of strong purifying selection acting on a subset of 12 genes required for meiosis and on the *rid* gene involved in RIP process indicates that these genes are maintained functional. In *P. roqueforti*, as in other species with economic importance, discovering the environmental conditions that promote or facilitate sexual reproduction would be extremely useful to allow classical strain improvement [11].

## Supporting Information

Figure S1 Gene trees reconstructed under a ML framework (see methods) for 12 meiotic genes (a-l). Divergences are indicated on branches. a) DMC1; b) MSH5; c) mus50; d) mutL; e) RAD21; f) RAD51; g) RAD54; h) rec8; i) ski8; j)protein required for establishment and maintenance of sister chromatid cohesion; k) MSH4; l) SPO11.
(DOC)

Figure S2 Color coded SELECTON Results for SPO11 sequence of *P. roqueforti.* 60% of the sites are evolving under purifying selection.
(DOC)

Table S1 Isolates of *Penicillium roqueforti* used in this study. FM numbers represent isolates provided by French stakeholders and their origin is confidential; LCP, CBS and IMI strains come from public collections; F isolates were obtained from 38 different cheeses coming from 14 diverse countries throughout the world (given as F followed by one to two digit values from A to 38 corresponding to the different cheeses, a dot and a final digit corresponding to the isolate number).
(DOC)

Table S2 Proteins of *Penicillium roqueforti* involved in meiosis and their homologs in *Neurospora crassa* as they were used for the BLASTP search.
(DOC)

Table S3 Isolates of *Penicillium roqueforti* used for population genetic analyses. FM numbers represent isolates provided by French stakeholders and their origin is confidential; LCP and IMI strains come from public collections. Four polymorphic markers were used: the MAT locus (MAT), the beta-tubulin (TUB) and the microsatellites PC4 and PC13. Each marker revealed polymorphism with two or three alleles each, coded 1, 2 and 3.
(DOCX)

Table S4 Proteins tested under SELECTON runs that are essential for meiosis. The number of sites evolving under purifying and positive selection is indicated as number of sites evolving under purifying or positive selection/total number of sites of the protein – Percentage.Proteins tested under SELECTON runs that are essential for meiosis.
(DOC)
