# Supplementary material for: Sex in Cheese: Evidence for Sexuality in the Fungus Penicillium roqueforti
Source: PLoS One. 2012 Nov 21;7(11):e49665. doi: 10.1371/journal.pone.0049665 (PMC3504111; doi:10.1371/journal.pone.0049665)
Supplement: Figure S1 — Gene trees reconstructed under a ML framework (see methods) for 12 meiotic genes (a-l). Divergences are indicated on branches. a) DMC1; b) MSH5; c) mus50; d) mutL; e) RAD21; f) RAD51; g) RAD54; h) rec8; i) ski8; j)protein required for establishment and maintenance of sister chromatid cohesion; k) MSH4; l) SPO11. (DOC) [file pone.0049665.s001.doc]

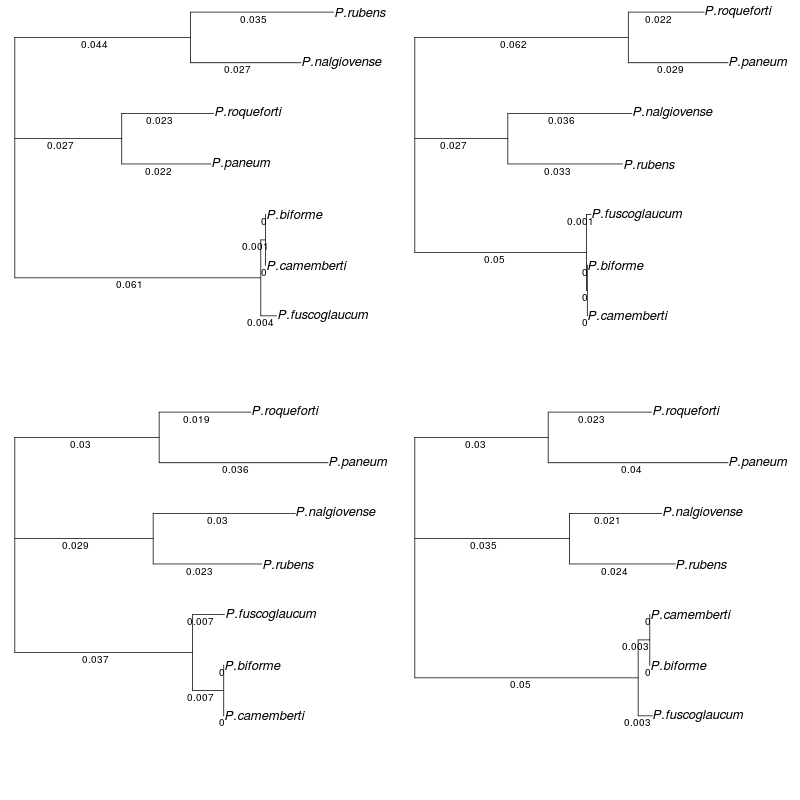
**Figure S1: Gene trees reconstructed under a ML framework (see methods) for 12 meiotic genes (a-l). Divergences are indicated on branches. a) DMC1 ; b) MSH5 ; c) mus50 ; d) mutL ; e) RAD21 ; f) RAD51 ; g) RAD54 ; h) rec8; i) ski8; j)protein required for establishment and maintenance of sister chromatid cohesion; k) MSH4; l) SPO11.**

(b)

(a)

(c)

(d)


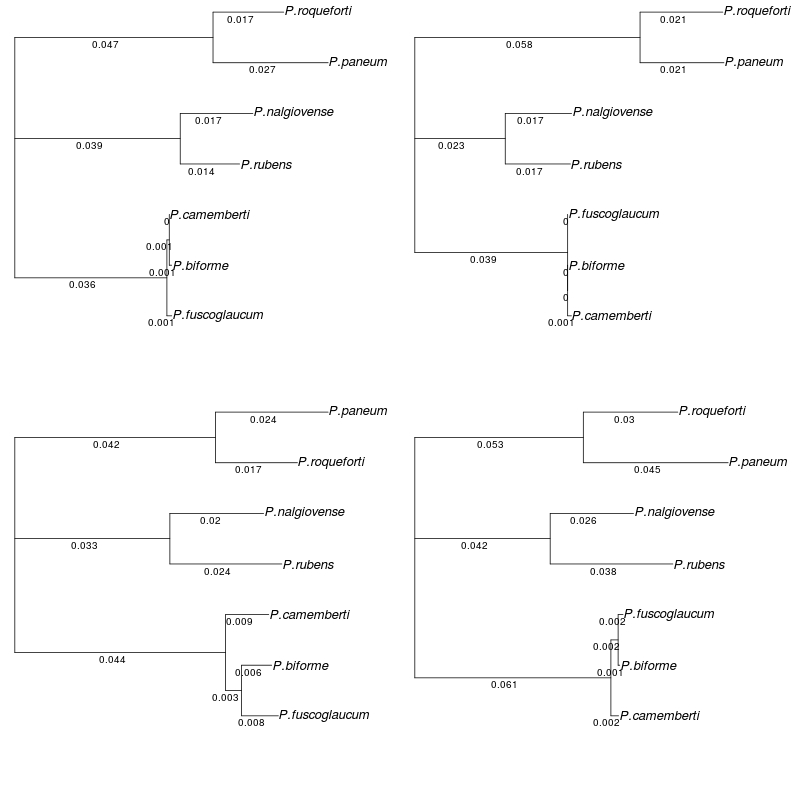


(e)

(f)

(g)

(h)


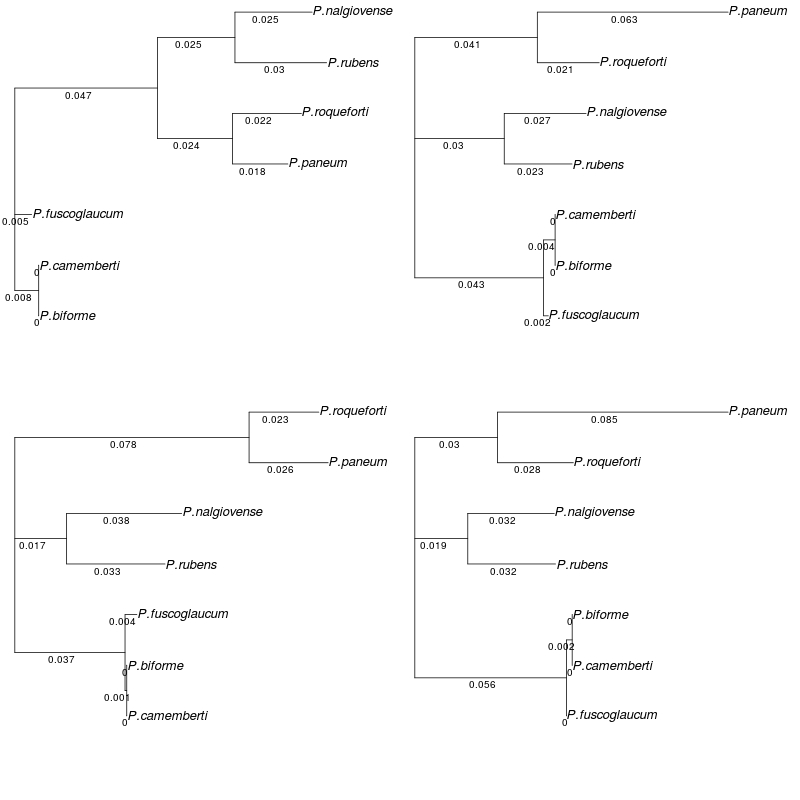


(l)

(k)

(j)

(i)
