# Supplementary material for: Sex in Cheese: Evidence for Sexuality in the Fungus Penicillium roqueforti
Source: PLoS One. 2012 Nov 21;7(11):e49665. doi: 10.1371/journal.pone.0049665 (PMC3504111; doi:10.1371/journal.pone.0049665)

**Figure S2: Color coded SELECTON Results for SPO11 sequence of *P. roqueforti.* 60% of the sites are evolving under purifying selection.**


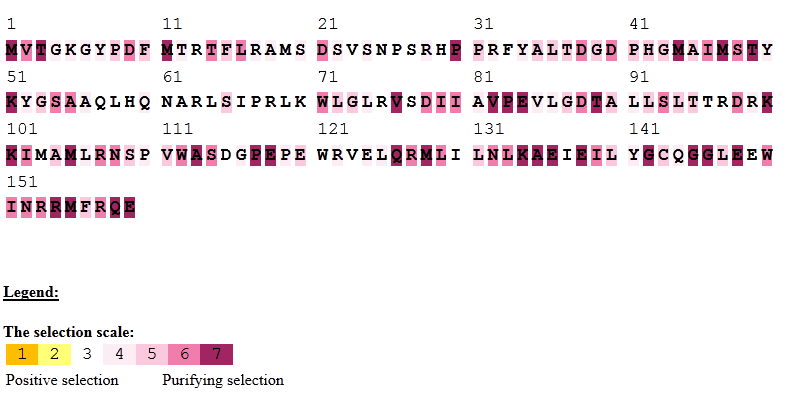

Supplement: Figure S2 — Color coded SELECTON Results for SPO11 sequence of P. roqueforti. 60% of the sites are evolving under purifying selection. (DOC) [file pone.0049665.s002.doc]
