# Supplementary material for: Sex in Cheese: Evidence for Sexuality in the Fungus Penicillium roqueforti
Source: PLoS One. 2012 Nov 21;7(11):e49665. doi: 10.1371/journal.pone.0049665 (PMC3504111; doi:10.1371/journal.pone.0049665)
Supplement: Table S1 — Isolates of Penicillium roqueforti used in this study. FM numbers represent isolates provided by French stakeholders and their origin is confidential; LCP, CBS and IMI strains come from public collections; F isolates were obtained from 38 different cheeses coming from 14 diverse countries throughout the world (given as F followed by one to two digit values from A to 38 corresponding to the different cheeses, a dot and a final digit corresponding to the isolate number). (DOC) [file pone.0049665.s003.doc]

Table S1: Isolates of *Penicillium roqueforti* used in this study. FM numbers represent isolates provided by French stakeholders and their origin is confidential; LCP, CBS and IMI strains come from public collections; F isolates were obtained from 38 different cheeses coming from 14 diverse countries throughout the world (given as F followed by one to two digit values from A to 38 corresponding to the different cheeses, a dot and a final digit corresponding to the isolate number).

|  | Isolate number | Substrate | Origin | MAT 1-1 | MAT 1-2 | |  |
| --- | --- | --- | --- | --- | --- | --- | --- |
| FM collection | FM 015 | Cheese environment | France | - | | + | |
|  | FM016 | Cheese environment | France | - | | + | |
|  | FM 037 | Cheese environment | France | - | | + | |
|  | FM 156 | Cheese environment | France | + | | - | |
|  | FM 157 | Cheese environment | France | + | | - | |
|  | FM 158 | Cheese environment | France | + | | - | |
|  | FM 159 | Cheese environment | France | + | | - | |
|  | FM 160 | Cheese environment | France | + | | - | |
|  | FM 162 | Cheese environment | France | - | | + | |
|  | FM 163 | Cheese environment | France | - | | + | |
|  | FM 164 | Gorgonzola cheese | France | - | | + | |
|  | FM 165 | Cheese environment | France | + | | - | |
|  | FM 167 | Cheese environment | France | + | | - | |
|  | FM 170 | Cheese environment | France | - | | + | |
|  | FM 171 | Cheese environment | France | + | | - | |
|  | FM 172 | Cheese environment | France | + | | - | |
|  | FM 173 | Cheese environment | France | + | | - | |
|  | FM 174 | Cheese environment | France | + | | - | |
|  | FM 175 | Cheese environment | France | + | | - | |
|  | FM 176 | Cheese environment | France | + | | - | |
|  | FM 177 | Cheese environment | France | + | | - | |
|  | FM 178 | Cheese environment | France | + | | - | |
|  | FM 179 | Cheese environment | France | - | | + | |
|  | FM 211 | Cheese environment | France | - | | + | |
|  | FM 215 | Cheese environment | France | - | | + | |
|  | FM 216 | Cheese environment | France | + | | - | |
|  | FM 217 | Cheese environment | France | + | | - | |
|  | FM 218 | Cheese environment | France | - | | + | |
|  | FM 219 | Cheese environment | France | + | | - | |
|  | FM 220 | Cheese environment | France | - | | + | |
|  | FM 221 | Cheese environment | France | - | | + | |
|  | FM 222 | Cheese environment | France | + | | - | |
|  | FM 223 | Cheese environment | France | + | | - | |
|  | FM 224 | Cheese environment | France | - | | + | |
|  | FM 225 | Cheese environment | France | - | | + | |
|  | FM 263 | Cheese environment | France | - | | + | |
|  | FM 315 | Cheese environment | France | + | | - | |
|  | FM 316 | Cheese environment | France | - | | + | |
|  | FM 317 | Cheese environment | France | + | | - | |
|  | FM 320 | Cheese environment | France | + | | - | |
|  | FM 358  FM 359 | Gorgonzola cheese | France | - | | + | |
|  |  | Gorgonzola cheese | France | - | | + | |
|  | FM 360 | Gorgonzola cheese | France | - | | + | |
| Public collections | LCP 75.146 | Roquefort cheese | France | - | | + | |
|  | LCP 50.148 | Brewery atmosphere | Unknown | + | | - | |
|  | LCP 64.1883 | Cheese | Unknown | - | | + | |
|  | LCP 88.2492 | Unknown | Unknown | + | | - | |
|  | LCP 93.2939 | Packing brioche | France | - | | + | |
|  | LCP 93.3676 | Stewed fruit | France | - | | + | |
|  | LCP 96.3914 | Stewed fruit | France | - | | + | |
|  | LCP 97.3969 | Stewed fruit | France | - | | + | |
|  | LCP 97.4111 | Wood | France | + | | - | |
|  | LCP 98.4180 | Strawberry sorbet | France | - | | + | |
|  | LCP 07.5419 | Fridge inner wall | France | + | | - | |
|  | LCP 07.5420 | Fridge inner wall | France | - | | + | |
|  | LCP 07.5421 | Fridge inner wall | France | + | | - | |
|  | LCP 11.5885 | Silage | France | + | | - | |
|  | CBS 449.78 | Cheddar cheese | Unknown | - | | + | |
|  | IMI 024313T | Roquefort cheese | Unknown | + | | - | |
| LUBEM Brest collection (directly isolated from various blue cheeses) | F1.1 | Blue cheese | Canada | - | | + | |
|  | F2.1 | Blue cheese | Canada | - | | + | |
|  | F3.1 | Blue cheese | Canada | - | | + | |
|  | F4.7 | Blue cheese | Canada | + | | - | |
|  | F5.2 | Fourme d'Ambert | France | + | | - | |
|  | F5.3 | Fourme d'Ambert | France | - | | + | |
|  | F6.3 | Gorgonzola | Italy | - | | + | |
|  | F7.1 | Gorgonzola | Italy | - | | + | |
|  | F7.3 | Gorgonzola | Italy | - | | + | |
|  | F8.1 | Gorgonzola | Italy | - | | + | |
|  | F9.4 | Fourme d'Ambert | France | - | | + | |
|  | F9.5 | Fourme d'Ambert | France | - | | + | |
|  | F10.1 | Bleu d'Auvergne | France | + | | - | |
|  | F10.2 | Bleu d'Auvergne | France | + | | - | |
|  | F10.3 | Bleu d'Auvergne | France | + | | - | |
|  | F10.5 | Bleu d'Auvergne | France | + | | - | |
|  | F11.5 | Carré Aurillac | France | - | | + | |
|  | F12.1 | Pigme | France | - | | + | |
|  | F12.2 | Pigme | France | - | | + | |
|  | F12.5 | Pigme | France | + | | - | |
|  | F13.1 | Carré Auvergne | France | - | | + | |
|  | F13.2 | Carré Auvergne | France | + | | - | |
|  | F13.3 | Carré Auvergne | France | - | | + | |
|  | F13.4 | Carré Auvergne | France | - | | + | |
|  | F14.1 | Gorgonzola | Argentina | - | | + | |
|  | F14.3 | Gorgonzola | Argentina | - | | + | |
|  | F14.5 | Gorgonzola | Argentina | - | | + | |
|  | F14.6 | Gorgonzola | Argentina | - | | + | |
|  | F15.1 | Blue cheese | Brazil | - | | + | |
|  | F15.3 | Blue cheese | Brazil | - | | + | |
|  | F15.4 | Blue cheese | Brazil | - | | + | |
|  | F16.1 | Picon Hoja | Spain | - | | + | |
|  | F16.6 | Picon Hoja | Spain | + | | - | |
|  | F17.1 | Peña Santa | Spain | + | | - | |
|  | F17.2 | Peña Santa | Spain | + | | - | |
|  | F18.1 | Tresviso | Spain | - | | + | |
|  | F18.2 | Tresviso | Spain | - | | + | |
|  | F19.1 | Peral | Spain | - | | + | |
|  | F20.1 | Cabrales | Spain | - | | + | |
|  | F20.4 | Cabrales | Spain | - | | + | |
|  | F21.1 | Blue cheese | Spain | + | | - | |
|  | F22.1 | Blue cheese | Netherlands | - | | + | |
|  | F22.2 | Blue cheese | Netherlands | - | | + | |
|  | F22.5 | Blue cheese | Netherlands | - | | + | |
|  | F23.1 | Blue cheese | Netherlands | - | | + | |
|  | F23.3 | Blue cheese | Netherlands | - | | + | |
|  | F24.2 | Blue cheese | Netherlands | - | | + | |
|  | F25.1 | Blue cheese | Netherlands | - | | + | |
|  | F25.6 | Blue cheese | Netherlands | - | | + | |
|  | F26.2 | Blue cheese | Netherlands | - | | + | |
|  | F26.3 | Blue cheese | Netherlands | - | | + | |
|  | F27.1 | Blue cheese | USA | + | | - | |
|  | F28.1 | Blue cheese | Latvia | - | | + | |
|  | F28.2 | Blue cheese | Latvia | - | | + | |
|  | F28.3 | Blue cheese | Latvia | + | | - | |
|  | F29.1 | Blue cheese | Denmark | - | | + | |
|  | F29.3 | Blue cheese | Denmark | - | | + | |
|  | F30.1 | Blue cheese | Poland | - | | + | |
|  | F31.2 | Blue cheese | Latvia | - | | + | |
|  | F32.1 | Blue cheese | Denmark | - | | + | |
|  | F33.1 | Blue cheese | Germany | - | | + | |
|  | F34.1 | Blue cheese | Estonia | - | | + | |
|  | F35.1 | Blue cheese | Germany | - | | + | |
|  | F36.1 | Blue cheese | Germany | - | | + | |
|  | F36.5 | Blue cheese | Germany | - | | + | |
|  | F37.1 | Blue cheese | Germany | - | | + | |
|  | F37.4 | Blue cheese | Germany | - | | + | |
|  | F38.1 | Blue cheese | Germany | - | | + | |
