# Supplementary material for: Sex in Cheese: Evidence for Sexuality in the Fungus Penicillium roqueforti
Source: PLoS One. 2012 Nov 21;7(11):e49665. doi: 10.1371/journal.pone.0049665 (PMC3504111; doi:10.1371/journal.pone.0049665)
Supplement: Table S2 — Proteins of Penicillium roqueforti involved in meiosis and their homologs in Neurospora crassa as they were used for the BLASTP search. (DOC) [file pone.0049665.s004.doc]

Table S1: Proteins of *Penicillium roqueforti* involved in meiosis and their homologs in *Neurospora crassa* as they were used for the BLASTP search.

| **Protein name** | ***Neurospora crassa* protein** | | ***P. roqueforti* protein** |
| --- | --- | --- | --- |
|
| **MEIOSIS** | | | |
| **DSB formation and processing** | | | |
| Meiosis-specific topoisomerase | NCU01120 | *spo11* | Proq06g075600 |
| Meiotic recombination protein | NCU03517 | *ski8* | Proq02g027210a |
| DEAD/DEAH box DNA helicase involved in CO (MER3) | NCU09793 |  | Proq14g113440a |
| Splicing factor 3b subunit 4 involved in formation of DSBs (MRE2) | NCU04182 |  | Proq12g102610 |
| Mutagen sensitive-23 involved in meiotic DSBs repair (MER11) | NCU08730 | *mus-23* | Proq01g000660 |
| Ultraviolet-sensitive-6 (RAD50) | NCU00901 | *usv6* | Proq14g113800 |
| DNA repair protein, component of the Mre11 complex | NCU04329 |  | Proq01g012390 |
| **Single strand invasion** | | | |
| Strand exchange protein DMC1 | AAA34571 Sc |  | Proq01g017270 |
| Strand exchange protein (RAD51) | NCU02741 | *mei3* | Proq02g030240a |
| Strand exchange protein | NCU04275 | *mus-11* | Proq04g057580a |
| DNA-dependent ATPase | NCU11255 | *rad-54* | Proq14g113180 |
| Replication factor-A protein 1 | NCU03606 | *rpa-1* | Proq01g017750a |
| Replication factor-A protein 2 | NCU07717 |  | Proq01g000310 |
| Strand exchange protein Rad55p | NCU08806 |  | Proq01g007390 |
| DNA repair protein rhp57 (Rad57p) | NCU01771 |  | Proq01g010730a |
| **DNA damage checkpoint** | | | |
| Genome integrity checkpoint protein | NCU00274 |  | Proq01g014980 |
| Cell cycle checkpoint protein RAD24 | NCU00517 |  | Proq02g026950 |
| Related to DNA repair protein RAD17 | NCU00942 |  | Proq05g071800 |
| **Proteins involved in CO** | | | |
| DNA mismatch repair enzyme related to Mlh1 | NCU08309 | *mutL* | Proq02g034420 |
| DNA mismatch repair protein related to Mlh3 | NCU05385 |  | Proq01g005120 |
| DNA mismatch repair ATPase (MutS ortholog 4) | NCU10895 | *msh4* | Proq04g062300 |
| DNA mismatch repair MutS family | NCU09384 | *msh5* | Proq02g031860 |
| RecQ family helicase SGS1 | NCU08598 | *mus-19* | Proq03g055880 |
| Meiosis specific protein | NCU10836 |  | None |
| Nucleotide-excision repair | NCU07440 | *mus-38* | Proq04g063810a |
| DNA excision repair protein Rad2 | NCU07498 |  | Proq03g043750 |
| **Synaptonemal complex** | | | |
| Histone H2A.Z | NCU05347 |  | Proq01g023810a |
| Histone H2A | NCU02437 |  | Proq07g083390 |
| Structural maintenance of chromosome: SMC protein | NCU09065 |  | Proq04g064030 |
| Structural maintenance of chromosome: SMC protein | NCU02402.5 |  | Proq07g083300a |
| Exonuclease | NCU06089 |  | Proq07g079330 |
| Kinase involved in chromosome segregation: casein kinase I isoform delta | NCU00685 |  | Proq03g049640a |
| Binds to damaged DNA during nucleotide excision repair | NCU07542 | *rad23* | Proq01g007070a |
| UvrD/REP helicase double-strand break repair via homologous recombination | NCU04733 | *mus-50* | Proq03g044840 |
| **Mismatch repair** | | | |
| DNA mismatch repair protein msh-2 | NCU02230 | *msh-2* | Proq03g050510 |
| DNA mismatch repair protein Msh3 | NCU08115 | *msh-3* | Proq01g014820 |
| DNA mismatch repair protein msh6 | NCU08135 | *msh-6* | Proq24g127740 |
| DNA mismatch repair protein | NCU08020 |  | Proq03g054450 |
| Protein involved in mismatch repair and in meiotic recombination | NCU09373 |  | Proq11g099690 |
| **Resolution of recombination intermediates** | | | |
| Protein involved in DNA reoair and recombination | NCU04047 |  | Proq09g091680 |
| Crossover junction endonuclease mus-81 | NCU07457 | *mus-81* | Proq03g048670a |
| GIY-YIG catalytic domain-containing protein | NCU01236 |  | Proq01g022360 |
| DNA topoisomerase I | NCU09118 |  | Proq04g065610a |
| DNA topoisomerase 2 | NCU06338 |  | Proq14g111790 |
| DNA topoisomerase 3-beta | NCU00081 |  | Proq03g044400 |
| **Nonhomologous end joining** | | | |
| Ku70 protein | NCU08290 | *mus-51* | Proq06g074400a |
| Ku80 protein | NCU00077 | *mus-52* | Proq03g047950a |
| DNA repair and recombination protein ligase MUS53 | NUC06264 | *mus-53* | Proq02g034030 |
| **Other** | | | |
| Protein of unknown function required for meiotic recombination | NCU04415 |  | Proq01g013720a |
| **COHESION** | | | |
| **Adherin** | | | |
| Subunit of cohesin loading factor | NCU05250 |  | Proq13g108260 |
| **Chromosome cohesion** | | | |
| Cohesin complex subunit | NCU01323 |  | Proq07g081820 |
| Chromosome segregation protein SudA | NCU07554 |  | Proq03g053170 |
| Cohesin complex subunit required for sister chromatid cohesion | NCU01247 |  | Proq02g038570 |
| Double-strand-break repair protein rad21 required for recombination | NCU03291 | *rad-21* | Proq13g108760 |
| Rec8 protein | NCU03190 |  | Proq04g063180 |
| Protein required for establishment and maintenance of sister chromatid cohesion | NCU00242 |  | Proq03g049550 |
| **Separin** | | | |
| Caspase-like protease involved in sister chromatid separation | NCU00205 |  | Proq06g077380 |
| **Condensins** | | | |
| Nuclear condensin complex subunit Smc2 | NCU07679 |  | Proq01g002850 |
| Nuclear condensin complex subunit Smc4 | NCU09063 |  | Proq07g079530 |
| Condensin complex component cnd1 | NCU09297 |  | Proq07g082960a |
| Nuclear condensin complex subunit 3 | NCU06216 |  | Proq01g023530 |
| **CHROMOSOME SEGREGATION** | | | |
| Spindle pole body component | NCU04535 |  | Proq14g113300 |
| Kinetochore-associated HEC/Ndc80p family protein | NCU03899 |  | Proq01g013590 |
| Chromosome segregation protein | NCU07984 |  | Proq16g117170 |
| Chromosome segregation in meiosis protein 3 | NCU01858 |  | Proq16g116730 |
| Kinesin | NCU04581 |  | Proq03g052910a |
| Alpha tubulin involved in nuclear migration along microtubules | NCU09132 |  | Proq04g061720a |
| Gamma-tubulin chain | NCU03954 | *tbg* | Proq01g012190 |
| Tubulin alpha-2 | NCU09468 | *tba-2* | Proq04g061720a |
| Ubiquitin-conjugating enzyme | NCU09731 | *ms-8* | Proq14g111500a |
| **ANAPHASE-PROMOTING COMPLEX** | | | |
| Anaphase-promoting complex/cyclosome subunit (APC1) | NCU05901 |  | Proq07g082780 |
| Anaphase-promoting complex subunit Apc5 | NCU01963 |  | Proq07g082150 |
| Anaphase-promoting complex subunit Cut9 | NCU01377 |  | Proq01g023080a |
| WD-repeat containing protein slp1 | NCU02616 |  | Proq10g097610 |
| Anaphase-promoting complex subunit CDC23 | NCU01174 |  | Proq01g023220 |
| Nuclear protein bimA | NCU00213 |  | Proq04g066190 |
| Anaphase-promoting complex subunit 10 | NCU08731 |  | Proq25g128590 |
| Cell cycle regulatory protein | NCU01269 |  | Proq07g081790 |
| WD domain-containing protein | NCU01572 |  | Proq05g068280 |
| **TRANSCRIPTION FACTOR AND GENE REGULATION** | | | |
| Meiosis-specific transcription factor | NCU09915 |  | Proq04g059340 |
| Histone-lysine N-methyltransferase | NCU06266 |  | Proq02g033960 |
| Transcription elongation factor S-II | NCU02563 |  | Proq01g003550 |
| Subunit of a complex involved in repression of meiotic sporulation | NCU00388 |  | Proq03g047430 |
| SNF2-family ATP dependent chromatin remodeling factor snf21 | NCU06488 |  | Proq01g020800 |
| **SIGNAL TRANSDUCTION** | | | |
| Ca/CaM-dependent kinase-1 | NCU09123 |  | Proq03g053930a |
| Protein kinase involved in cell cycle checkpoint in response to DNA damage | NCU00274 |  | Proq01g014980 |
| Protein kinase gsk3 involved in tranducing signal to enter in meiosis | NCU04185 |  | Proq12g102830a |
| Serine threonine protein kinase involved in cell cycle regulation | NCU07378 |  | Proq11g097950 |
