# Supplementary material for: Sex in Cheese: Evidence for Sexuality in the Fungus Penicillium roqueforti
Source: PLoS One. 2012 Nov 21;7(11):e49665. doi: 10.1371/journal.pone.0049665 (PMC3504111; doi:10.1371/journal.pone.0049665)
Supplement: Table S3 — Isolates of Penicillium roqueforti used for population genetic analyses. FM numbers represent isolates provided by French stakeholders and their origin is confidential; LCP and IMI strains come from public collections. Four polymorphic markers were used: the MAT locus (MAT), the beta-tubulin (TUB) and the microsatellites PC4 and PC13. Each marker revealed polymorphism with two or three alleles each, coded 1, 2 and 3. (DOCX) [file pone.0049665.s005.docx]

Table S3: Isolates of *Penicillium roqueforti* used for population genetic analyses. FM numbers represent isolates provided by French stakeholders and their origin is confidential; LCP and IMI strains come from public collections. Four polymorphic markers were used: the MAT locus (MAT), the beta-tubulin (TUB) and the microsatellites PC4 and PC13. Each marker revealed polymorphism with two or three alleles each, coded 1, 2 and 3.

| Isolate number | MAT | Tub | PC4 | PC13 |  | Isolate number | MAT | Tub | PC4 | PC13 |
| --- | --- | --- | --- | --- | --- | --- | --- | --- | --- | --- |
| FM 015 | 2 | 1 | 1 | 1 |  | LCP 75.146 | 2 | 1 | 1 | 1 |
| FM016 | 2 | 1 | 1 | 1 |  | LCP 50.148 | 1 | 2 | 2 | 2 |
| FM 037 | 2 | 2 | 1 | 1 |  | LCP 64.1883 | 2 | 2 | 2 | 2 |
| FM 156 | 1 | 1 | 2 | 2 |  | LCP 88.2492 | 1 | 2 | 2 | 2 |
| FM 157 | 1 | 1 | 2 | 2 |  | LCP 93.2939 | 2 | 2 | 2 | 2 |
| FM 158 | 1 | 1 | 2 | 2 |  | LCP 93.3676 | 2 | 1 | 2 | 2 |
| FM 159 | 1 | 1 | 2 | 2 |  | LCP 96.3914 | 2 | 3 | 2 | 2 |
| FM 160 | 1 | 1 | 2 | 2 |  | LCP 97.3969 | 2 | 1 | 2 | 2 |
| FM 162 | 2 | 1 | 1 | 1 |  | LCP 97.4111 | 1 | 2 | 2 | 2 |
| FM 163 | 2 | 1 | 1 | 1 |  | LCP 98.4180 | 2 | 1 | 2 | 2 |
| FM 164 | 2 | 1 | 1 | 1 |  | LCP 07.5419 | 1 | 1 | 2 | 2 |
| FM 165 | 1 | 1 | 2 | 2 |  | LCP 07.5420 | 2 | 2 | 2 | 2 |
| FM 167 | 1 | 1 | 2 | 2 |  | LCP 07.5421 | 1 | 1 | 2 | 2 |
| FM 170 | 2 | 1 | 1 | 1 |  | IMI 024313T | 1 | 2 | 2 | 2 |
| FM 171 | 1 | 1 | 2 | 2 |  |  |  |  |  |  |
| FM 172 | 1 | 1 | 2 | 2 |  |  |  |  |  |  |
| FM 173 | 1 | 1 | 2 | 2 |  |  |  |  |  |  |
| FM 174 | 1 | 1 | 2 | 2 |  |  |  |  |  |  |
| FM 175 | 1 | 1 | 2 | 2 |  |  |  |  |  |  |
| FM 176 | 1 | 1 | 2 | 2 |  |  |  |  |  |  |
| FM 177 | 1 | 1 | 2 | 2 |  |  |  |  |  |  |
| FM 178 | 1 | 1 | 2 | 2 |  |  |  |  |  |  |
| FM 179 | 2 | 1 | 1 | 1 |  |  |  |  |  |  |
| FM 211 | 2 | 2 | 1 | 1 |  |  |  |  |  |  |
| FM 215 | 2 | 1 | 1 | 1 |  |  |  |  |  |  |
| FM 216 | 1 | 1 | 2 | 2 |  |  |  |  |  |  |
| FM 217 | 1 | 1 | 2 | 2 |  |  |  |  |  |  |
| FM 218 | 2 | 2 | 1 | 1 |  |  |  |  |  |  |
| FM 219 | 1 | 1 | 2 | 2 |  |  |  |  |  |  |
| FM 220 | 2 | 1 | 1 | 1 |  |  |  |  |  |  |
| FM 221 | 2 | 1 | 1 | 1 |  |  |  |  |  |  |
| FM 222 | 1 | 1 | 2 | 2 |  |  |  |  |  |  |
| FM 223 | 1 | 2 | 1 | 1 |  |  |  |  |  |  |
| FM 224 | 2 | 1 | 1 | 1 |  |  |  |  |  |  |
| FM 225 | 2 | 2 | 1 | 1 |  |  |  |  |  |  |
| FM 263 | 2 | 2 | 1 | 1 |  |  |  |  |  |  |
| FM 315 | 1 | 1 | 2 | 2 |  |  |  |  |  |  |
| FM 316 | 2 | 1 | 1 | 1 |  |  |  |  |  |  |
| FM 317 | 1 | 2 | 2 | 2 |  |  |  |  |  |  |
