# Supplementary material for: Sex in Cheese: Evidence for Sexuality in the Fungus Penicillium roqueforti
Source: PLoS One. 2012 Nov 21;7(11):e49665. doi: 10.1371/journal.pone.0049665 (PMC3504111; doi:10.1371/journal.pone.0049665)
Supplement: Table S4 — Proteins tested under SELECTON runs that are essential for meiosis. The number of sites evolving under purifying and positive selection is indicated as number of sites evolving under purifying or positive selection/total number of sites of the protein – Percentage.Proteins tested under SELECTON runs that are essential for meiosis. (DOC) [file pone.0049665.s006.doc]

Table S4: Proteins tested under SELECTON runs that are essential for meiosis. The number of sites evolving under purifying and positive selection is indicated as number of sites evolving under purifying or positive selection / total number of sites of the protein – Percentage.Proteins tested under SELECTON runs that are essential for meiosis.

| Protein name | *P. roqueforti* protein | Nb of sites evolving under: | |
| --- | --- | --- | --- |
|  |  | Purifying selection | Positive selection |
| Double-strand breaks formation and processing | | | |
| Meiosis-specific topoisomerase SPO11 | Proq06g075600 | 99/159 – 62% | 0 |
| Meiotic recombination protein (ski8) | Proq02g027210a | 272/305 –89% | 1/305 – 0.3% |
| Single strand invasion | | | |
| Strand exchange protein DMC1 | Proq01g017270 | 313/327 – 96% | 0 |
| Strand exchange protein (RAD51) | Proq02g030240a | 324/344 – 94% | 2/344 – 0.6% |
| DNA-dependent ATPase (RAD54) | Proq14g113180 | 739/816 – 91% | 3/816 – 0.4% |
| Proteins involved in crossover | | | |
| DNA mismatch repair enzyme related to Mlh1 (mutL) | Proq02g034420 | 649/752 – 86% | 7/752 – 0.9% |
| DNA mismatch repair ATPase (MSH4) | Proq04g062300 | 660/817 – 81% | 0 |
| DNA mismatch repair MutS family (MSH5) | Proq02g031860 | 717/869 – 83% | 7/869 – 0.8% |
| Synaptonemal complex | | |  |
| UvrD/REP helicase double-strand break repair via homologous recombination (MUS50) | Proq03g044840 | 871/976 – 89% | 15/976 – 1.5% |
| Chromosome cohesion | | | |
| Double-strand-break repair protein rad21 required for recombination | Proq13g108760 | 539/586 – 92% | 3/586 – 0.5% |
| Rec8 protein | Proq04g063180 | 507/648 – 78% | 8/648 – 1.2% |
| Protein required for establishment and maintenance of sister chromatid cohesion | Proq03g049550 | 314/359 – 88% | 3/359 – 0.8% |
